# Is Primatology an Equal-Opportunity Discipline?

**DOI:** 10.1371/journal.pone.0030458

**Published:** 2012-01-17

**Authors:** Elsa Addessi, Marta Borgi, Elisabetta Palagi

**Affiliations:** 1 Unità di Primatologia Cognitiva, Istituto di Scienze e Tecnologie della Cognizione, Consiglio Nazionale delle Ricerche, Rome, Italy; 2 Centro Interdipartimentale Museo di Storia Naturale e del Territorio, Università di Pisa, Calci, Pisa, Italy; 3 Dipartimento di Biologia Cellulare e Neuroscienze, Istituto Superiore di Sanità, Rome, Italy; Georgia State University, United States of America

## Abstract

The proportion of women occupying academic positions in biological sciences has increased in the past few decades, but women are still under-represented in senior academic ranks compared to their male colleagues. Primatology has been often singled out as a model of “equal-opportunity” discipline because of the common perception that women are more represented in Primatology than in similar fields. But is this indeed true? Here we show that, although in the past 15 years the proportion of female primatologists increased from the 38% of the early 1990s to the 57% of 2008, Primatology is far from being an “equal-opportunity” discipline, and suffers the phenomenon of “glass ceiling” as all the other scientific disciplines examined so far. In fact, even if Primatology does attract more female students than males, at the full professor level male members significantly outnumber females. Moreover, regardless of position, IPS male members publish significantly more than their female colleagues. Furthermore, when analyzing gender difference in scientific productivity in relation to the name order in the publications, it emerged that the scientific achievements of female primatologists (in terms of number and type of publications) do not always match their professional achievements (in terms of academic position). However, the gender difference in the IPS members' number of publications does not correspond to a similar difference in their scientific impact (as measured by their H index), which may indicate that female primatologists' fewer articles are of higher impact than those of their male colleagues.

## Introduction

Women's representation in science has increased in the past few decades [1]–[3]. This growth has been more pronounced in the fields of life sciences (including health and biomedical sciences), social sciences and psychology, while in the most math-intensive fields (i.e., engineering, physics, mathematics, chemistry, economics, and computer science), women's progress has been much less dramatic [1], [3]. For life sciences, in 1970 13% of PhDs were awarded to women, whereas this percentage increased to 52% today. In other disciplines, the percentages of PhDs awarded to women are even higher (57% of all MD degrees, 71% of PhDs in Psychology and 77% of DVMs) [3].

The increase in women's proportion of full-time tenured or tenure-track faculty appears to reflect the rising inflow of female graduates entering the fields of science and engineering in recent years [4]. Despite this influx, in many disciplines women continue to be under-represented in senior academic ranks, holding a larger share of assistant professor positions than of associate or full professor positions [1], [2]. According to the last report of European Commission on Gender Equality, in 2006 only 18% of European full professors were women (11% in the fields of science and engineering), although women made up more than half of the university population [2]. In 2008 in Germany only 11% of full professors and 7% of the directors of the prestigious Max Planck Institutes were women [5]. Likewise, in the period 1990–2004, although in the USA there were more female than male graduate students in biological and medical sciences, only 19% of women held a tenure-track position at NIH [6], [7]. A similar disparity between the ratio of men and women in independent faculty positions was found in most academic institutions across the USA [1]. The imbalance in men's and women's representation is even more extensive in Japan. Despite of slight advances in recent years, Japan is still far below the European Union and US averages, with women representing only 12.4% of the research community, overwhelmingly concentrated in the most junior positions (graduate students, postdocs, and technicians) [8]. The situation is worsened by the so-called ‘*koza*’ system, which places huge power in the hands of full professors (usually men), who have almost complete control over funding and hiring decisions [9].

Women's under-representation in high academic ranks and in prestigious institutions may prevent them from having access to resources necessary to produce high-quality work, and this factor, in turn, affects their productivity, in terms of articles published at each professional stage [3]. From an analysis of the publications and citations count (source: ISI Web of Knowledge) for 39 female and 129 male scientists in the fields of Ecology and Evolutionary Biology who held faculty positions in Australian and British universities [10], men published about 40% more scientific articles than women and this difference appeared very early in their career (starting 2 years after the first publication). Similarly, from an analysis of the patenting and publication record (Source: Science Citation Index) of 4227 life scientists earning a PhD between 1967 and 1995 with a post-PhD five-year experience of publication, it emerged that women patent much less than men (about 5% of the women and 13% of the men held patents). This finding is affected by other differences between male and female academics, such as (i) number of papers, (ii) amount of NIH grants, and (iii) number of papers coauthored with researchers in industry, all factors that are systematically higher in men than in women. However, even holding constant productivity, social network, scientific field, and employer characteristics, female life scientists patent at about 40% of the rate of their male counterparts [11].

The most likely reasons of women's under-representation in senior academic ranks are not biological differences [12] or an open discrimination of female researchers in funding, hiring, and publishing, although there are conflicting findings on this point [3], [13], [14]–[19], but rather gender differences in family and parental responsibilities, resources, and interests. Women occupy less prestigious positions providing fewer resources likely because of free choices and/or biology and society constraints, such as the necessity to defer careers and/or limit job searches to raise children, follow partners' career moves, and care for elderly parents [3], [5], [7], [11], [20]. Furthermore, women may not like to push themselves forward, to narrow down their spectrum of interests for career, to exert power and to make unpopular decisions when occupying leading positions [5] and show a relative lack of confidence in applying for potentially prestigious positions [5], [7], [20]. A survey commissioned by the US Congress and conducted by the US National Research Council in 2004 and 2005 on almost 500 departments in six fields (Biology, Chemistry, Civil Engineering, Electrical Engineering, Mathematics, and Physics) indicates that the proportion of women's applications for tenure-track positions was lower than the percentage of PhDs awarded to women for the same discipline [4]. Similarly, in the late 90s many prestigious fellowships (such as the Human Frontier Science Program and the Wellcome Trust) had significantly fewer female than male applicants [21].

In the National Science Foundation longitudinal analysis of the academic career paths of men and women, family characteristics, specifically marital status and the presence of children, were found to be related to women's chances to be employed in tenure-track positions [1]. Women are particularly disadvantaged early in their careers: the transition from postdoctoral fellow to faculty is a period during which a worrying number of women leave academic research [7]. Individual choices, such as deferring careers during childrearing years, might also explain, at least in part, why women spend significantly longer time as assistant professors than do men [4]. Interestingly, in a very recent analysis of the impact of the scientific career on family life, although nearly twice as many women as men reported having fewer children than desired because they pursued a science career, family factors impede talented young scientists of both sexes from persisting to research positions in academic science [22].

Primatology has been singled out as a model of “equal-opportunity” discipline mainly because of the enormous interest of the media that made the work of the three most popular female primatologists (Dian Fossey, Jane Goodall and Biruté Galdikas) into spectacles [23], thus leading to the common perception that women are more represented and influential in Primatology than in similar fields. The three most popular primatologists are in fact women who have been a source of inspiration for many female (and male) field primatologists (the so-called “National Geographic effect” [24]). Primatology has undoubtedly changed over the past sixty years of its existence, by including an increasing proportion of women, which have made a greater than average impact on this discipline [24]–[26]. Both Haraway [27] and Hrdy [28] found that women were disproportionately represented among primatologists compared to their representation in other sciences. The thorough analysis carried out by Fedigan [24] showed that there was a significantly higher proportion of women in Primatology than in analogous biological sciences, such as Ornithology, Mammalogy, and Benthology. In fact, in 1991 women made up 48% of the membership of the American Society of Primatologists (ASP) and 38% of the International Primatological Society (IPS), but only about 25%, on average, of the members of the analogous biological disciplines examined.

However, there were not significantly more women in Primatology than in its parental disciplines (such as Psychology, Anthropology, and Animal Behavior). Thus, the perception that there was a larger proportion of women in Primatology than in related sciences is valid relative to other biological sciences, but not valid in comparison to other behavioral sciences from which Primatology has originated [29]. The only study, to our knowledge, that took into account primatologists' scientific productivity (a possibly more reliable measure than membership in professional societies) was a survey of the publications of Brazilian primatologists in Current Primate References over the period 1985–1996 [30]. It emerged that men published more than women, although this analysis was limited to Brazilian scientists [30].

In all the life science disciplines examined so far [4]–[7], [13], [31], women are over-represented among graduate students and their number progressively decreases when proceeding to the top levels of the academic career, becoming under-represented (in comparison to male colleagues) at the full professor level. However, up to now, no study has yet investigated whether Primatology suffers the phenomenon of the “glass ceiling”. Thus, the first goal of our research is to evaluate the relative number of male and female primatologists (i.e., active members of the IPS in the year 2008) at each level of the academic career (from graduate students to full professors). Our second goal is to assess male and female primatologists' scientific productivity (measured in terms of number of publications) and their impact on the scientific community (measured by the H-index).

## Methods

We obtained information on country, gender, and position for 820 out of 1366 IPS members in good standing in the year 2008 from the database published on the IPS website. As in Fedigan's study [24], individuals were categorized as men or women on the basis of their first names. For African and Asian authors' names, we consulted colleagues who were familiar with the languages and members in question. We omitted from the analysis the 44 members who had ambiguous first names and we used the Chi square test to compare the total number of male and female IPS members both for all the 55 countries with at least one IPS member and for the countries with more than 10 members (Table 1).

**Table 1:** For each country, total number of female and male IPS members, number of female and male IPS members holding an academic position, number of female and male IPS members holding a non academic position, and chi square values.

Country	F	M	Chi square	M academics	F academics	Chi square	M non academics	F non academics	Chi square
**Brazil**	7	8	0.07, NS	7	7	-	1	0	-
**Canada**	21	9	4.8, p = 0.03	7	21	7.0, p = 0.008	2	0	-
**France**	6	5	0.09, NS	4	5	0.11, NS	1	1	-
**Germany**	35	33	0.06, NS	24	31	0.89, NS	9	4	1.9, NS
**Italy**	17	8	3.2, NS	6	16	4.5, p = 0.033	2	2	-
**Japan**	10	34	13.1, p<0.001	33	9	13.7, p<0.001	1	1	-
**Spain**	3	9	3.0, NS	9	2	4.5, p = 0.035	0	1	-
**United Kingdom**	68	37	9.1, p = 0.002	33	65	7.0, p = 0.008	4	3	0.14, NS
**United States**	246	146	25.5, p<0.001	122	198	18.1, p<0.001	24	47	7.45, p = 0.006

The table reports only the countries with more than 10 IPS members.

We omitted from the analysis also the 501 members for which position was unknown and we grouped in two different categories (academics and non-academics) the IPS members holding academic and non-academic positions, respectively. Specifically, non-academics included curators, zoo keepers, research assistants, zoo directors, and technicians. Academics were further grouped in six positions, that - depending on the terminology employed in different countries and/or research institutions – included: (i) master students, PhD students and graduate students (hereafter, graduate students), (ii) post-docs, (iii) assistant professors, lecturers, research scientists (hereafter, assistant professors), (iv) associate professors, readers, senior research scientists (hereafter, associate professors), (v) full/emeriti professors and research directors (hereafter, full professors), and (vi) department/institute directors (hereafter, academic directors).

We used the Chi square test to compare the number of IPS members holding academic versus non academic positions and, within academics, we assessed which positions (graduate students, post-docs, assistant professors, associate professors, full professors, and academic directors) were most represented by using the standardized residual analysis. We used the Chi square test also to compare the number of men and women within non-academics and academics, respectively, and the number of men and women within each most represented academic position.

For each IPS member holding one of the most represented academic positions (graduate students, assistant professors, and full professors, see Results), we counted the number of publications listed on the Primatelit database up to 2008. We chose only national/international journal papers with or without impact factor. We excluded abstracts, proceedings, book chapters, and books. The number of publications was normalized on each member's years of scientific activity calculated from the year of publication of the first paper (as found in Primatelit) up until 2008. Then, we split each member's publications in four categories according to the order of the IPS member's name in the list of authors (single name, first name, middle name, and last name). We carried out a mixed-model ANOVA on the total number of publications with gender and position as between-subject factors and type of publication as a within-subject factor. We used the Tukey HSD test for post-hoc comparisons.

Finally, for assistant professors and full professors, we obtained the H-indices [32] by using the free software “Publish or Perish”. In order to avoid the bias due to the different number of years of activity of each member we limited our search to the years 2000–2008. We used the t-test for independent samples to compare the H-indices of male and female assistant and full professors, respectively.

## Results

### Gender distribution of the IPS members

Among the IPS members there was an overall significant prevalence of women over men, both when considering all the 55 countries with at least one IPS member (M = 42.7%, F = 57.3%, χ_1_^2^ = 17.6, p<0.001) and when limiting the analysis to the nine countries with more than 10 members (M = 41.2%, F = 58.8%, χ_1_^2^ = 21.9, p<0.001) (Table 1).

Most of the IPS members are academics (academics: 83.9%, non-academics: 16.1%; Chi square test: χ^2^_1_ = 377.9, p<0.001). When considering academics (see Methods), women were significantly more than men (F = 58.0%, M = 42.0%, χ_1_^2^ = 17.6, p<0.001), whereas when considering non-academics there was no significant difference between the number of men and women (F = 53.8%, M = 46.2%, χ_1_^2^ = 0.76, p = 0.38, NS). When analysing the data for each country separately, there was a prevalence of women over men among IPS members holding an academic position in Canada, Italy, United Kingdom, and United States, whereas for Japan and Spain the opposite pattern was found, and for Brazil, France, and Germany there was no significant difference. Only for the IPS members based in the United States there was a prevalence of women over men also among non academics (Table 1).

**Figure 1:**
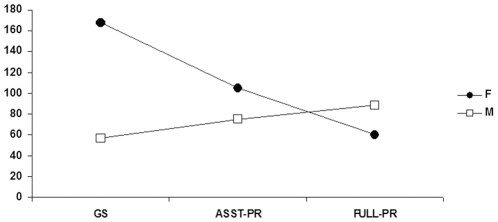
Number of male and female IPS member for each academic position. Number of male (white square) and female (black dots) graduate students (GS), assistant professors (ASST-PR) and full professors (FULL-PR) in the IPS in the year 2008.

Among academics, there was a prevalence of graduate students (32.6%), assistant professors (26.2%), and full professors (21.7%) (standardized residual analysis, graduate students: 109.3, p<0.01; assistant professors: 65.3, p<0.01; full professors: 34.3, p<0.01), whereas associate professors, post-docs, and academic directors accounted for 11.5%, 5.9% and 2.2% of the academics, respectively (standardized residual analysis, associate professors: −35.7, p<0.01; post-docs: −73.7, p<0.01; academic directors: −99.7, p<0.01). Women were significantly more than men among graduate students and assistant professors (graduate students: F = 74.6%, M = 25.4%, χ_1_^2^ = 54.0, p<0.001; assistant professors: F = 58.3%, M = 41.7%, χ_1_^2^ = 5.0, p = 0.02), whereas among full professors the opposite held true (F = 40.3%, M = 59.7%, χ_1_^2^ = 5.6, p = 0.02) (Figure 1). We could not perform the latter analyses for each country because for most of the countries there were sample size limitations.

### Publications

The ANOVA revealed a significant effect of gender (men: 1.31±0.07, women: 0.90±0.04; F_1,496_ = 4.75, p = 0.03) and position (graduate students: 0.47±0.04, assistant professors: 1.17±0.07, full professors: 1.56±0.09; F_2,496_ = 48.03, p<0.0001) on the total number of publications, but no significant interaction between gender and position (F_2,496_ = 1.17, p = 0.31, NS). Thus, regardless of position, men published more than women and post-hoc tests showed that, regardless of gender, full professors published more than graduate students (Tukey HSD test, p<0.0001) and assistant professors (Tukey HSD test, p<0.0001) and that assistant professors published more than graduate students (Tukey HSD test, p<0.0001).

Moreover, there was a significant difference between the four types of publications (single-name: 0.20±0.01, first-name: 0.37±0.02, middle-name: 0.27±0.01, last-name: 0.24±0.02; F_3,1488_ = 18.12, p<0.0001) and significant interactions between type of publication and position (F_6,1488_ = 8.44, p<0.0001), and type of publication and gender (F_3,1488_ = 2.96, p = 0.03). However, there was no significant interaction between type of publication, gender, and position (F_6,1488_ = 1.25, p = 0.30, NS).

When considering male primatologists, full professors published more than the other professional categories (full professors vs. graduate students: Tukey HSD test, p<0.0001; full professors vs. assistant professors: Tukey HSD test, p = 0.02; Table 2). This difference was due to the number of last-name articles, which was higher for male full professors than for both graduate students (Tukey HSD test, p<0.0001; Table 2) and assistant professors (Tukey HSD test, p<0.01; Table 2). Instead, male assistant professors published more than graduate students regardless of publication type (Tukey HSD test, p<0.001; Table 2).

**Table 2:** Mean number (± standard error) of the number of publications per year (total, single-name, first-name, middle-name, last-name) for graduate students, assistant professors, and full professors according to gender.

	Females	Males
Graduate students	N = 134	N = 49
Total	0.46±0.05	0.51±0.09
Single-name	0.07±0.02	0.09±0.04
First-name	0.21±0.04	0.28±0.06
Middle-name	0.13±0.02	0.08±0.03
Last-name	0.04±0.01	0.06±0.03

Assistant professors	N = 98	N = 72
Total	1.11±0.08	1.25±0.14
Single-name	0.21±0.03	0.20±0.03
First-name	0.42±0.04	0.45±0.06
Middle-name	0.30±0.04	0.30±0.04
Last-name	0.18±0.03	0.30±0.06

Full professors	N = 61	N = 88
Total	1.34±0.13	1.71±0.13
Single-name	0.24±0.03	0.37±0.04
First-name	0.37±0.04	0.38±0.03
Middle-name	0.34±0.05	0.36±0.03
Last-name	0.39±0.06	0.59±0.07

When considering female primatologists, full professors published more than graduate students but not than assistant professors (Tukey HSD test, p<0.0001; Table 2). This difference was due to the number of last-name papers (Tukey HSD test, p<0.0001; Table 2). Regardless of publication type, female assistant professors published more than female graduate students (Tukey HSD test, p<0.0001; Table 2), whereas no significant difference was found between female full professors and female assistant professors (Tukey HSD test, p = 0.64, NS; Table 2).

### H index

The H-indices did not significantly differ between male and female assistant professors (H-index: men: 6.04±0.66, women: 5.28±0.40, t_168_ = 1.04, p = 0.30, NS) and between male and female full professors (H-index: men: 7.80±0.75, women: 7.15±0.82, t_147_ = −0.57, p = 0.57, NS).

## Discussion

In 2008, female IPS members were 57%, one third more than 15 years ago [24]. Thus, there has been a steady increase in the women's representation in Primatology that followed the trend of other life science disciplines [3]. The prevalence of women over men held true only for the IPS members holding an academic position, whereas among the IPS members holding a non-academic position there was a similar proportion of male and female members. However, when analyzing the gender distribution for each academic position, a predominance of female members was evident only for graduate students and assistant professors, whereas for full professors there was an opposite gender distribution.

When considering the nine countries with more than 10 IPS members, there was a prevalence of women in Canada, United Kingdom, and United States, a prevalence of men in Japan and no significant difference in gender distribution in all the other countries examined. Similarly, there was a prevalence of women among academics in Canada, Italy, United Kingdom, and United States, a prevalence of men in Japan and Spain, and no significant difference in Brazil, France, and Germany. Only in the United States there was a prevalence of women over men also among non-academics. Our analysis, however, cannot consider how the specific cultural context of each country influences the dynamic of scientific careers (i.e., the gender distribution for each academic position) because of sample size limitations.

Overall, since among the IPS members the number of female graduate students is more than twice that of males, our data indicate that Primatology attracts especially female students. Despite this, among full professors male members significantly outnumber females. Thus, Primatology suffers the phenomenon of “glass ceiling” as all the other life science disciplines examined so far [5]–[7], [13], [21], [31]. It is unlikely that the difference between the number of female assistant and full professors can be explained as a cohort effect. In fact, from the results of the 1998 Membership Survey of the American Association of Physical Anthropology [31], a relevant discipline because it also includes primatologists, it emerged that between 1970 and 1990 there was an important increase in the number of PhDs awarded to women (35% in 1970s, 65% in 1990s), in the number of women obtaining a tenure-track job (30% in 1970s, 55% in 1990s) and obtaining tenure (29% in 1970s, 54% in 1990s), but there was a much lower increase in the number of women being promoted to the rank of full professor (18% in 1970s, 32% in 1990s).

A further confirmation of the above finding comes from the analysis of the gender difference in the number of publications. Regardless of position, male IPS members publish significantly more than their female colleagues. Thus, as in Ecology and Evolutionary Biology [10], a striking difference in scientific productivity emerges very early in the academic career of primatologists.

When analyzing gender difference in scientific productivity in relation to the type of publication (i.e., in terms of the author's name order: first name, middle name, last name, and single name), it emerged that male full professors have a significantly higher number of last-name publications than male assistant professors and graduate students. In contrast, female full professors have a significantly higher number of last-name publications than female graduate students, but not than female assistant professors. Since the last name in a publication is often that of the scientist who coordinated the study, we argue that the number of senior-coordinated publications of female primatologists does not always predict their academic position, and that, as it is evident from the literature, it may take quite a long time for female primatologists to be promoted to senior academic ranks [3], [4], [6], [11], [31]. A review of the careers of North-American scientists and engineers has found that women are promoted more slowly than men [33] and similar results have been obtained for the same fields also more recently [20], [21].

Alternatively, the lack of a significant difference in the number of senior-coordinated publications between female assistant professors and female full professors might be due to the fact that in past years more female primatologists were promoted to full professor with a relatively little number of senior-coordinated publications, whereas in more recent years this phenomenon has changed probably because Primatology has become a more competitive discipline than in the past. Interestingly, there was no significant difference in the number of last-name publications between male and female full professors possibly because women increase their rate of publication after childrearing years or men decrease it as they reach the top levels of the academic career. Future studies should address this issue in order to assess which of these two hypotheses hold true.

However, the striking gender difference in terms of number of publications found among the IPS members does not reflect a difference in scientific impact. In fact, both at the assistant and full professor level, male and female primatologists do not significantly differ in their H index, that is - to date - the most used measure of scientific production impact. Since the H index is affected also by the author's number of publications, the lack of such a difference may indicate that female primatologists produce articles of higher scientific impact than expected for their lower productivity, as it has been proposed for Ecology and Evolutionary Biology [10]. Similar results have been obtained from the analysis of the publication record (Source: Science Citation Index) of 4227 life scientists earning a PhD over 30 years. Although men published more than their female colleagues, their research impact – as measured by the mean citation count per article – did not significantly differ [11]. Similarly, when considering the publications from 1999 to 2006 of the 1998 applicants to the European Molecular Biology Organization's (EMBO) fellowships, it emerged that even awarded women published significantly less than men, but impact factor did not significantly differ between awarded men and women [13]. Likewise, from the comparison of data for 57 male and 48 female academics leading Library and Information Science departments, it emerges that men publish more than women, but there are no significant gender differences in the number of citations [34]. Nonetheless, some of the limitations of the H-index might have affected both our findings and those of the above cited studies, namely the difficulty to obtain the complete output of scientists with very common names, or the problem of self-citations, which can increase a scientist's H-index (although the effect of self-citations on the H-index is quite small since only those with a number of citations higher than the H-index are relevant) [35].

Thus, our findings overturn the view that Primatology is an “equal-opportunity” discipline, as it has been often claimed by the media. As happens in other life science disciplines [4], [6], [7], Primatology attracts more female students than males, but the gender distribution pattern switches at the top levels of the academic career. Moreover, regardless of position, female primatologists have a lower scientific productivity than male colleagues and the women's scientific achievements in terms of number of last-name publications do not match their professional achievements in terms of academic position. Nonetheless, the similarity between the male and female primatologists' H index may indicate that female primatologists' fewer articles are of higher impact than those of their male colleagues. In conclusion, in Primatology women are still under-represented at the top levels of the academic career. If this under-representation of women in the senior academic ranks is due to a glass ceiling or to a higher family load experienced by women than by men, is still an open question in all the disciplines examined so far, with different studies leading to contrasting results. Further research is needed to disentangle this issue.
